# Neural hypersensitivity to pleasant touch in women remitted from anorexia nervosa

**DOI:** 10.1038/s41398-018-0218-3

**Published:** 2018-08-16

**Authors:** Amanda Bischoff-Grethe, Christina E. Wierenga, Laura A. Berner, Alan N. Simmons, Ursula Bailer, Martin P. Paulus, Walter H. Kaye

**Affiliations:** 1 Department of Psychiatry, University of California, San Diego, CA USA; 2 VA San Diego Healthcare System, San Diego, CA USA; 3 Division of Biological Psychiatry, Department of Psychiatry and Psychotherapy, Medical University of Vienna, Vienna, Austria; 4 Laureate Institute for Brain Research, Tulsa, Oklahoma USA

## Abstract

Interoception, or the sensing and integration of bodily state signals, has been implicated in anorexia nervosa (AN), given that the hallmark symptoms involve food restriction and body image disturbance. Here we focus on brain response to the anticipation and experience of affective interoceptive stimuli. Women remitted from AN (RAN; *N* = 18) and healthy comparison women (CW; *N* = 26) underwent a pleasant affective touch paradigm consisting of gentle strokes with a soft brush administered to the forearm or palm during functional neuroimaging. RAN had a lower brain response relative to CW during anticipation of touch, but a greater response when experiencing touch in the right ventral mid-insula. In RAN, this reduced anticipatory response was associated with higher levels of harm avoidance. Exploratory analyses in RAN also suggested that lower response during touch anticipation was associated with greater body dissatisfaction and higher perceived touch intensity ratings. This reduced responsivity to the anticipation of pleasant affective interoceptive stimuli in association with higher harm avoidance, along with an elevated response to the experience of touch, suggests an impaired ability in AN to predict and interpret incoming physiological stimuli. Impaired interoception may thus impact one’s sense of self, thereby supporting observations of disturbed body image and avoidance of affective and social stimuli. Therapeutic approaches that help AN to better anticipate and interpret salient affective stimuli or improve tolerance of interoceptive experiences may be an important addition to current interventions.

## Introduction

Anorexia nervosa (AN) is a debilitating eating disorder characterized by extreme dietary restriction, a relentless drive for thinness, and body image disturbance, resulting in a dangerously low body weight^1^. Despite advances in the field, the etiology of self-starvation and disturbed experience of one’s body weight and shape in AN remains poorly understood. Individuals with AN have shown an altered brain response to physiological sensations including taste^2–5^, hunger signaling^6^, stomach distention^7^, heartbeat and gut attention^8^, aversive breathing load^9^, and pain^10^. These impairments suggest that interoception, defined as the ability to sense and process the physiological condition of one’s body, contributes to AN pathophysiology^11^. Moreover, interoceptive awareness supports affective functioning^12^ and, when perturbed, contributes to body image distortion^13,14^. However, it is unknown whether pleasant affective interoceptive experiences are also altered in AN, which may impact approach motivation and the drive to eat.

The experience and interpretation of bodily sensations provide both a mechanism for establishing emotions (e.g., pleasure or disgust) and an awareness that guides behavior^11^. Research demonstrating altered interoception in AN has focused on unpleasant (e.g., thermal pain), neutral (e.g., heart rate), or symptom-specific (e.g., gut monitoring, taste, hunger) interoceptive signaling. However, affective touch, a pleasant interoceptive stimulus^15,16^ that acts on both sensory and emotional systems via different afferent fibers in the skin (e.g., palmar Aβ-fibers that relay sensory tactile information, C-fibers that convey sensory and hedonic information) to promote an awareness of one’s body, also helps guide behavior and social interactions and may be altered in AN^17–21^.

Both learning and adaptive behavior depend upon associations between anticipated and actual outcomes^22^. In AN, a mismatch between actual and anticipated interoceptive events may disrupt one’s experience of one’s body and induce avoidance behavior, such as dietary restriction and social isolation, similar to avoidance behaviors seen in individuals with high levels of anxiety^23,24^. This altered sensitivity to both anticipation and receipt of physiological, sensory, and affective stimuli has often been reported in AN^25–28^. Women with AN also experience difficulty distinguishing actual from anticipated interoceptive signals, such as feelings of fullness^7^, pain^29–31^, and heartbeat sensations^32,33^, supporting the involvement of these processes in AN.

The insula is a hub for the evaluation of interoceptive cues, having a pivotal role in the anticipation and processing of sensations, in order to guide behavior^11^. The anterior insula codes interoceptive prediction error by signaling a mismatch between actual and anticipated bodily arousal, which in turn can elicit subjective anxiety and approach or avoidance behavior^11,24,34,35^. It projects to the ventral striatum (comprising the nucleus accumbens, rostroventral putamen, and ventromedial caudate)^36^, which is involved in identifying rewarding and emotionally significant stimuli^37^ to mediate goal-directed behaviors^38^. The ventral striatum projects back to the anterior insula^39,40^, thereby enabling the integration of anticipation with arousal. In particular, the more ventral aspects of the middle/anterior insula process social–emotional and sensorimotor information^41^, providing a mechanism for the integration of interoceptive stimuli with an emotional response^38^ that generates an action or decision^42^.

Neuroimaging findings suggest both ill and remitted AN show increased anterior insula response during anticipation, but reduced anterior insula response when experiencing pain^10^ and sweet taste^3,4^ compared with control women (CW), although the opposite pattern has been seen in response to aversive breathing loads^9^. AN also show reduced insula activation to viewing self-images^43^, and a greater insular response and higher satisfaction ratings when viewing thin self-images^44^ compared with CW. These data suggest that interoception in AN is dysregulated, with an impaired ability to anticipate, interpret, and integrate internal and external sensations. As interoception motivates goal-directed behavior^45^, an important question is whether AN experience positively valenced interoceptive stimuli differently than CW in circuits associated with motivation.

This is the first neuroimaging study to examine how women remitted from AN (RAN) differ from CW in their neural response in interoceptive and reward neurocircuitry during soft touch, a pleasant, non-disorder-specific, interoceptive stimulus, on the palm and forearm. We examined remitted AN participants to avoid the confounding effects of malnutrition on neural function. A well-validated soft touch task was used that has been shown to activate the insula and striatum^46–48^. We hypothesized that RAN would show an elevated response to the anticipation and a blunted response to the receipt of soft touch in the insula and striatum compared with CW, consistent with prior studies of pain^10^ and taste^3,49^, which would suggest a domain generalized interoceptive deficit. Differences in touch application site would inform the degree to which somatosensory (e.g., palm) versus affective (e.g., forearm) touch is implicated in AN. We also predicted that RAN with greater anxiety and harm avoidance (a construct comprising elements of anxiety, inhibition, and inflexibility)^50^ would show the most aberrant activation during anticipation and receipt of soft touch, consistent with studies showing associations between interoceptive dysregulation and anxiety^8,24,51^. Finally, we explored whether the anticipation and receipt of pleasant touch were associated with perceived pleasantness and intensity ratings in both groups, and, for RAN only, with body dissatisfaction, and past illness severity.

## Methods

### Participants

Eighteen RAN women (14 pure restricting type; 4 binge-eating/purging type, with regular purging but no binge-eating behavior) were compared with 26 CW. Remittance was defined^52^ as maintaining a weight above 85% of ideal body weight, regular menstrual cycles, and the absence of binge-eating, purging, and restrictive eating patterns for at least 1 year before the study^52^. RAN were recruited nationally and CW were recruited locally. Current and past psychiatric history were assessed using the MINI International Neuropsychiatric Interview by masters level assessors^53^. Participants also completed the State-Trait Anxiety Inventory^54^, the Temperament and Character Inventory^50^, the Beck Depression Inventory-II^55^, and the Eating Disorders Inventory-2 (EDI-2)^56^. Women were excluded from the study if they met diagnostic criteria for a current *DSM-IV* Axis I diagnosis, took psychotropic medication within 3 months before the study, had a history of alcohol or drug abuse, or dependence 3 months before study, were left-handed, or reported any medical or neurologic concerns contraindicative to magnetic resonance imaging (MRI). After providing subjects a complete description of the study, written informed consent was obtained. The University of California, San Diego Human Research Protections Program approved all procedures.

### Imaging procedures

#### Soft touch paradigm

Gentle strokes with a soft boar bristle brush (OXO International Ltd., NY) were administered on 4 cm-long regions of the skin by a trained research assistant. Stimulation occurred on either the ventral surface of the left forearm, a region believed to contain dense mechano-receptive C-fibers, or the palm, where these fibers are absent^57–59^. As in prior studies^46–48^, these regions were both pre-measured and pre-marked for consistency, and each soft brush stroke occurred at a velocity of 2 cm/s in a proximal to distal direction, standardized by an audio tone that was routed to the research assistant’s headphones. This velocity is within the optimal range (1–10 cm/s) for C-fiber stimulation and has been previously shown to activate the posterior insula^57^. The force applied was equal to the brush’s weight (8 oz).

**Fig. 1:**
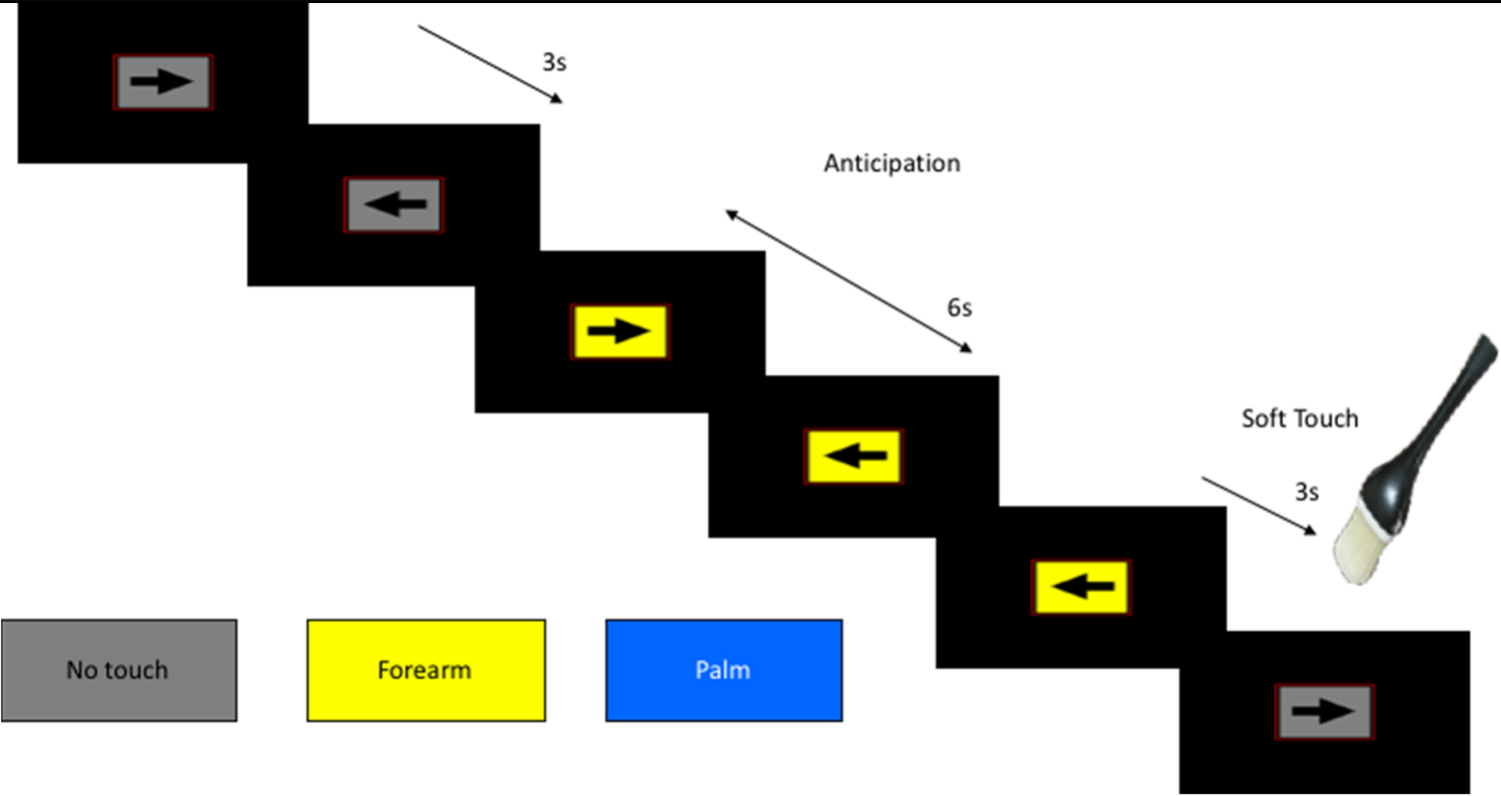
The soft touch continuous performance task. Participants were presented with either a left- or rightward pointing arrow on a gray background for 3 s and were asked to press the left or right button of a button box, using the index and middle finger of the right hand, which corresponded with the direction of the arrow. The arrow's background color indicated one of three conditions: 1) baseline, in which no stimulus was expected or administered (three consecutive arrow trials, or ~9 s duration); 2) anticipation of soft touch of the left forearm (yellow background, 6 s), for which the participant could expect a subsequent soft brushing of the forearm; and 3) anticipation of soft touch of the left palm (blue background, 6 s), which indicated the participant should expect a soft brushing of the palm. Following the anticipatory periods, the participant would experience a soft brush on either for forearm or palm (2 s)

Participants performed two task runs during functional MRI (Fig. 1). During each run, participants completed a continuous performance task, whereby they were presented with a left- or rightward pointing arrow on a gray rectangular background (3 s). Subjects were asked to press the left or right button of a button box using the index and middle fingers of the right hand, which corresponded to the direction of the arrow. The arrow’s background would change color to indicate one of three conditions as follows: (1) the baseline condition (gray background), in which no stimulus was expected or administered, and averaging 9 s (three consecutive arrow trials) in duration; (2) anticipation of soft touch of the left forearm (yellow background, 6 s), indicating the participant could expect with 100% likelihood a subsequent soft touch of the forearm; and (3) anticipation of soft touch of the left palm (blue background, 6 s), indicating the participant should expect a soft touch of the palm. Following the anticipatory periods, the soft touch condition would occur (3 s), whereby the brush was applied to the previously indicated location for the first 2 s of the trial. All participants were informed of the task structure and the meaning of the colored backgrounds before task performance. Across both runs, anticipation and soft touch occurred 20 times for each location (palm, forearm). Each run lasted 420 s.

Response accuracy and reaction time on the continuous performance task were recorded for all trials. Participants also completed pre- and post-functional MRI (fMRI) visual analog scale (VAS) questionnaires that rated soft touch of the forearm and palm, respectively, from “0—not at all” to “10—extremely” on pleasantness, unpleasantness, and intensity.

#### Image acquisition

Neuroimaging data were acquired using T2* weighted echo planar imaging (EPI) on a 3T General Electric Discovery MR 750 (Milwaukee, WI) (252 volumes, repetition time (TR) = 2 s, echo time (TE) = 30 ms, flip angle = 90°, field of view 24 cm, 64 × 64 matrix, 3.75 × 3.75 mm in-plane resolution, forty 3.0 mm ascending interleaved axial slices). High-resolution T1-weighted fast spoiled gradient echo (FSPGR) anatomical images (flip angle = 8°, 256 × 256 matrix, one hundred and seventy-two 1 mm sagittal slices, TR = 8.1 s, TE = 3.17 ms, 1 × 1 mm in-plane resolution) were acquired to permit activation localization and spatial normalization. EPI-based field maps corrected susceptibility-induced geometric distortions.

#### Image preprocessing

Functional images were preprocessed and analyzed using Analysis of Functional NeuroImages (AFNI)^60^ and FSL^61^ software. EPIs were slice-time corrected, motion-corrected, and aligned to high-resolution anatomical images using AFNI’s align_epi_anat.py^62^. Time points with isolated head movements not corrected by coregistration were censored. T1-weighted images were skull-stripped with FreeSurfer’s mri_watershed^63^ and registered to the MNI-152 atlas using affine transform followed by nonlinear refinement using FSL’s FLIRT and FNIRT^64,65^. Functional data were aligned to standard space, resampled to 3 mm isotropic voxels, and smoothed with a 4.2 mm FWHM Gaussian kernel. For each participant, AFNI’s 3dDeconvolve was used to determine activation related to the soft touch paradigm. Four task regressors (anticipation palm, anticipation forearm, soft touch palm, soft touch forearm) were convolved with a modified hemodynamic response function. Six motion regressors and five noise regressors of orders of polynomials trends (baseline, linear, quadratic, etc.) were included as covariates of no interest. Following deconvolution, the four task beta regressors were converted to percent signal change.

### Data analysis

#### Behavioral analysis

Group level statistical analyses were performed using the nlme package in R (http://www.r-project.org). Trial response accuracy and reaction time were recorded from the onset of arrow presentation. A linear mixed effects (LME) model examined reaction time differences, with group (CW, RAN) as the between-subject variable and Condition (anticipation, soft touch) and Location (palm, forearm) as the within-subjects variables. The VAS predictors for “pleasantness,” “unpleasantness,” and “intensity” were analyzed as dependent measures using a one-way analysis of variance (ANOVA) to test for group differences in subjective reports. A separate LME analysis determined whether there were any Group × Location interactions. An additional LME examined the VAS ratings, with group as the between-subject variable and Location as the within-subject variable. Finally, within-group Pearson’s product–moment correlations examined the relationship between intensity rating, and pleasantness and unpleasantness ratings as unpleasant experiences, which may be reported as feeling more intense^66^.

#### Regions of interest

Regions of interest (ROIs) were derived from the Harvard–Oxford atlas^67^. Two bilateral ROIs were defined: an insula ROI, which contained the insula in its entirety, and a striatum ROI that included the caudate, putamen, and nucleus accumbens. These ROIs were used as search regions for all fMRI analyses.

#### Neuroimaging analysis

Data were analyzed using a Group (CW, RAN) × Condition (anticipation, soft touch) × Location (palm, forearm) LME approach. For all analyses, subject was treated as a random effect, with Group, Condition, and Location as fixed effects. The spatial autocorrelation function (acf) option in AFNI’s 3dFWHMx estimated intrinsic smoothness. Minimum cluster sizes were calculated with AFNI’s 3dClustSim, in order to guard against false positives. For ROI analyses, a peak voxel of *p* < 0.01 with a cluster threshold of α < 0.025 (Bonferroni corrected for two ROIs) was required for significance. This approach employs non-Gaussian models and spatial acfs and is more robust than traditional methods^68^. Although a more stringent statistical threshold (e.g., *p* < 0.001) has been recommended^68^, a more liberal threshold is acceptable for smaller sample sizes, when the analysis is limited to a small number of ROIs^69^, or when event-related designs are used^70^. The required minimum cluster size was 270 μL (10 contiguous voxels) for each ROI. An exploratory whole brain analysis examined group differences in activation across the whole brain (peak voxel of *p* < 0.01, cluster threshold of *α* < 0.05, resulting in a minimum cluster size 837 μL [31 contiguous voxels]). For significant clusters, post-hoc analyses were conducted using *glht* from R’s multcomp package with Tukey’s all-pair comparisons, and the *p*-values were false discovery rate (FDR)^71^ adjusted.

#### Primary robust regression analyses

Voxelwise Huber robust regressions^72^ were conducted in R to examine the association of harm avoidance and trait anxiety with the mean percent signal change of the blood oxygen level dependent (BOLD) response. Within-group individual regressions were performed against the percent signal change for anticipation palm, anticipation forearm, soft touch palm, and soft touch forearm. As above, significant clusters were identified within each ROI search region using AFNI’s 3dClustSim for small volume correction with a peak voxel of *p* < 0.01. Results were Bonferroni corrected for two ROIs, two anxiety measures, and four touch task conditions (α < 0.003). To assess whether ROI-based clusters identified in the task-related LME analysis overlapped with those identified in the robust regression analyses of anxiety, we computed the intersection of the task-based clusters with those from the robust regression. As both maps include only significant clusters, the resultant overlap may also be considered statistically significant^73^.

#### Exploratory regression analyses

Exploratory Huber robust regression analyses examined the relationship between neural activation and subjective VAS ratings for pleasantness (measuring positive valence), unpleasantness, and intensity (measuring arousal), and, within the RAN group only, clinical variables for AN duration, lowest body mass index (BMI), months since last symptoms of AN (i.e., duration of remission), and EDI-2 Body Dissatisfaction using AFNI’s 3dClustSim for small volume correction with a peak voxel of *p* < 0.01. As these were exploratory, no Bonferroni correction was applied (α < 0.05). Finally, the overlap of significant clusters identified with exploratory robust regressions of clinical variables with the task-related LME analysis were also explored. Due to non-normal distributions, VAS predictors were natural log transformed and z-scored before regression. The EDI-2 Interoceptive Awareness subscale was not examined because most participants (both CW and RAN) scored 0 on this measure.

## Results

### Participant characteristics

CW and RAN did not differ significantly on current BMI (RAN mean = 21.8; CW mean = 21.7, *p* = 1.0), age (RAN mean = 26.3 years; CW mean = 26.3 years; *p* = 1.0), or years of education (RAN mean = 15.8; CW mean = 15.3; *p* = 0.5) (Supplemental Table 1). Groups also did not differ significantly on self-reported interoceptive awareness as measured by the EDI-2 (RAN mean = 0.7, CW mean = 0.2, *p* = 0.5). RAN reported greater levels of trait anxiety and harm avoidance than CW (*p*s < 0.001).

### VAS scales

One CW failed to complete the post-scan VAS ratings. One-way ANOVAs did not detect significant differences between groups for VAS pleasantness ratings to palm (Supplemental Table 2) or forearm (*p*s > 0.9) soft touch. In a separate LME analysis, no main effect of Group or Location, and no Group × Location interaction was detected for either pre-scan or post-scan pleasantness ratings (*p*s > 0.2). Groups also did not differ in VAS unpleasantness ratings to touch of the palm or forearm (*p*s > 0.2). However, RAN reported at post-scan that soft touch palm was more intense than CW, with most CW (*n* = 18) rating palm intensity at 0 on this measure (*p* = 0.02). RAN with higher intensity ratings also rated palm (*r* = 0.57, *p* = 0.01) or forearm (*r* = 0.50, *p* = 0.03) soft touch as more unpleasant. This was not seen in the CW group.

### Behavioral analyses

Four participants’ (three CW, one RAN) behavioral responses were lost due to equipment failure. Groups did not differ on continuous performance task accuracy (*p*s > 0.2). For reaction time, there was a significant main effect of Condition (*F*(1,114) = 5.7, *p* = 0.02), which suggested a slower response time during soft touch receipt compared to anticipation across all participants. No other main effects or interactions were significant (*p*s > 0.4).

### ROI analyses

#### Main effect of condition

**Fig. 2:**
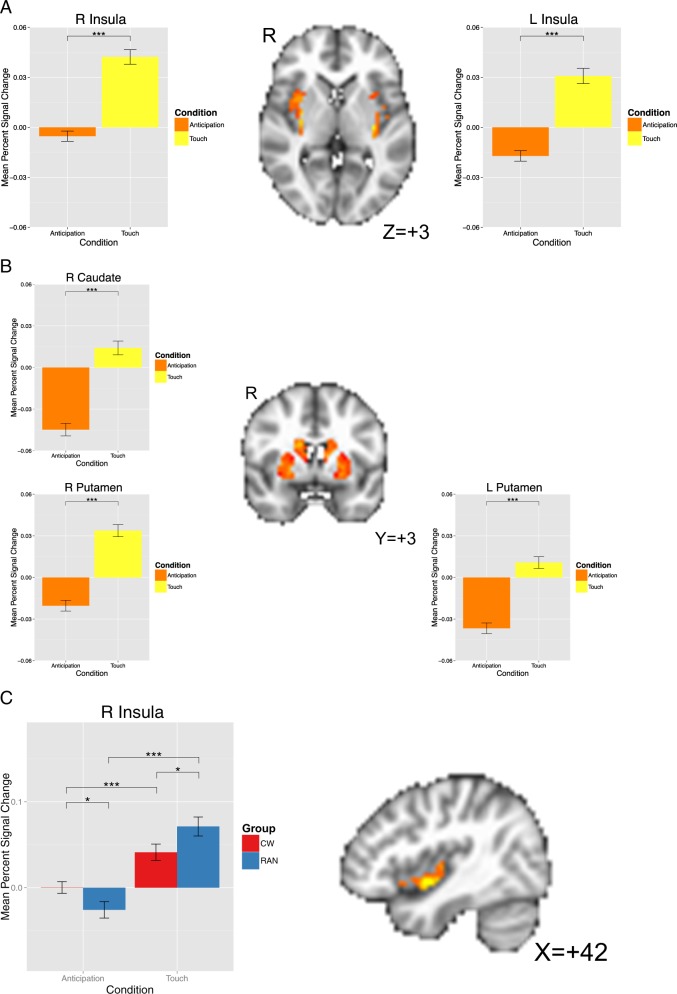
**A)** Bar plot showing a main effect of Condition (Anticipation, Soft Touch) within the bilateral insula.Overall, participants showed a greater BOLD response during soft touch relative to during anticipation. **B)** Bar plot showing a main effect of Condition within the bilateral dorsal striatum that included both the caudateand putamen. **C)** Bar plot showing significant Group (CW, RAN) x Condition (Anticipation, Soft Touch)10.1038/s41398-018-0218-3interactions during performance of the soft touch paradigm within the right ventral insula. While both groupsshowed greater BOLD response during soft touch relative to during anticipation, RAN had lower BOLDresponses during anticipation relative to CW, but higher BOLD responses during soft touch compared toCW. BOLD: blood oxygen level dependent; CW: healthy comparison women; RAN: women remitted fromanorexia nervosa; L: left; R: right. *p<0.05; ***p<0.005

Across both groups, both the bilateral insula, encompassing the anterior, middle, and posterior portions, and the dorsal striatum (centered in the putamen and including the dorsal caudate) showed a greater response during soft touch receipt than during anticipation (Table 1, Fig. 2a, b).

**Table 1:** LMEs analysis results within the bilateral insula demonstrating an interaction of Group (CW, RAN) by Condition (anticipation, soft touch) and a main effect of Condition for the soft touch paradigm

							Post Hoc comparisons		
Region	L/R	Volume (voxels)	*X*	*Y*	*Z*	*F*-value	Comparison	*z*	p(FDR)
Main effect of condition									
Insula	L	186	− 35	− 9	3	26.34	Soft Touch > Anticipation	4.36	< 0.001
	R	169	35	− 2	5	21.69	Soft Touch > Anticipation	4.23	< 0.001
Dorsal putamen	L	423	− 21	0	6	28.96	Soft Touch > Anticipation	4.59	< 0.001
	R	168	27	− 3	2	34.99	Soft Touch > Anticipation	4.86	< 0.001
Dorsal caudate	R	224	14	8	14	27.31	Soft Touch > Anticipation	4.47	< 0.001
Group × Condition									
Ventral mid-insula	R	15	41	− 1	− 8	11.83	CW: Soft Touch > Anticipation	3.53	< 0.001
							Anticipation: CW > RAN	1.97	0.049
							Soft Touch: RAN > CW	2.27	0.028
							RAN: Soft Touch > Anticipation	6.98	< 0.001

Note: Although both groups had a greater response during touch receipt vs. anticipation in the right ventral mid-insula, RAN had lower responses during anticipation but greater responses during soft touch compared to CW. Center of mass coordinates reported in MNI space. Small volume correction was determined with Monte-Carlo simulations (via AFNI’s 3dClustSim) to guard against false positives. Post-hoc analyses were conducted using glht from the multcomp package in R to calculate general linear hypotheses using Tukey’s all-pair comparisons, and *p*-values were FDR adjusted. *CW* healthy comparison women, *L* left, *LME* linear mixed effects, *R* right, *RAN* women remitted from anorexia nervosa.

#### Group × Condition

A significant group × condition interaction in the right ventral mid-insula revealed that RAN had a lower BOLD response during anticipation, but a greater BOLD response during soft touch than CW (Table 1, Fig. 2c). No interactions were detected within the striatum.

There were no significant findings within the insula or striatum for any other interaction (i.e., Group × Condition × Location, Group × Location, Condition × Location), or for the main effect of either Group or Location.

### Exploratory whole brain analyses

#### Condition

There was a significant main effect of Condition, with multiple clusters throughout frontal, temporal, parietal, and occipital regions showing a greater response during soft touch receipt relative to anticipation. Only the right middle frontal gyrus showed the opposite relationship, with greater activation during soft touch anticipation relative to receipt (Supplemental Table 3).

#### Location

There was a main effect of Location within the right postcentral gyrus, with all participants showing a greater response to the palm compared with the forearm.

#### Group × Condition

As in the ROI analysis, a Group × Condition interaction was detected within the right ventral mid-insula and extending into the superior temporal gyrus, such that both groups showed a greater response during soft touch compared to anticipation, but this difference was more pronounced in the RAN group than the CW.

There were no significant findings for any other interaction (i.e., Group x Condition × Location, Group × Location, Condition × Location), or for the main effect of Group.

### Primary robust regression analyses

**Fig. 3:**
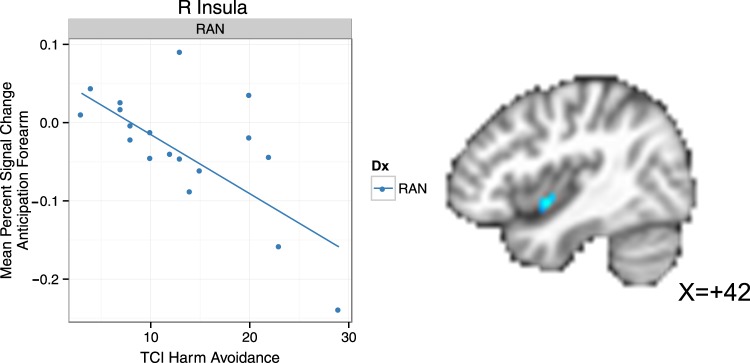
RAN [t=-4.59, p=0.002] with higher harm avoidance had lower BOLD response during anticipationof touch of the forearm in the right ventral mid-insula, as identified by Huber robust regression. BOLD: bloodoxygen level dependent; RAN: women remitted from anorexia nervosa; L: left; R: right; TCI: Temperament Character Inventory

RAN with higher harm avoidance scores had lower BOLD responses during forearm touch anticipation in right ventral mid-insula (Fig. 3, Table 2). Moreover, this harm-avoidance-associated cluster overlapped with the Group × Condition cluster, suggesting that harm avoidance may have had a role in the reduced anticipatory response in RAN. CW with higher harm avoidance scores had lower BOLD response in the right dorsal anterior insula during forearm soft touch receipt. There were no significant relationships with trait anxiety in either group, nor were there any significant relationships with anxiety measures in the striatum.

**Table 2:** Significant clusters identified by robust regression associating brain activity with harm avoidance within the insula

Event	Region	L/R	Volume (voxels)	*X*	*Y*	*Z*	*t*	*r*	*p*	Overlap (voxels)
CW										
Soft Touch Forearm	Insula	L	21	− 33	4	10	− 3.82	− 0.56	0.003	0
RAN										
Anticipation Forearm	Ventral mid-insula	R	9	42	0	− 7	− 4.59	− 0.68	0.002	6

Note: Coordinates are reported as the center of mass. Overlap refers to the number of voxels in the robust regression significant cluster which overlap with voxels in the Group × Condition interaction cluster [see Table 1]. CW healthy comparison women, L left, R right, RAN women remitted from anorexia nervosa.

### Exploratory robust regression analyses

#### VAS ratings

Analyses suggested that CW with higher pleasantness ratings tended to show higher BOLD responses in both the caudate and putamen during anticipation and receipt of soft touch, particularly on the forearm (Supplemental Table 4). In comparison, RAN with higher pleasantness ratings had greater BOLD responses in the caudate only to soft touch receipt. CW reporting greater intensity ratings demonstrated increased BOLD to both soft touch anticipation and receipt in the insula, particularly for the palm. In comparison, RAN with greater intensity ratings had lower BOLD responses during anticipation of soft touch forearm within the bilateral insula and dorsal striatum, but elevated BOLD response during soft touch palm in the bilateral ventral mid-insula and left putamen. There were no overlaps of task-related clusters with those associated with VAS ratings.

#### Clinical measures

RAN with lower historical BMIs had higher BOLD responses in the right ventral anterior insula, and lower BOLD responses in the dorsal mid-insula during soft touch forearm (Supplemental Table 4). RAN with longer illness durations also had greater BOLD responses during soft touch forearm in the left ventral anterior insula. These AN-severity-related clusters did not overlap with task-related clusters. RAN with higher body dissatisfaction also showed lower BOLD response during anticipation of soft touch forearm in the right ventral mid-insula, and this minimally (1 voxel) overlapped with the task-related cluster (Supplemental Fig. 1).

## Discussion

The present study suggests that AN is associated with altered neural signals that predict and interpret pleasant interoceptive stimuli. Relative to CW, RAN demonstrated a lower BOLD response in the right ventral mid-insula during anticipation of soft touch, but a greater right ventral mid-insula BOLD response during soft touch receipt compared with CW. These findings add to a growing literature, suggesting that neural preparation for and processing of interoceptive stimuli is disturbed in AN. Moreover, RAN with lower neural responses to touch anticipation were the most harm avoidant, and those with greater hyperactivation during touch receipt experienced pleasant interoceptive events more intensely. Finally, exploratory findings showing associations between higher body dissatisfaction with reduced ventral mid-insula response during touch anticipation suggest this may also contribute to an altered subjective experience of one’s body. These findings suggest disrupted interoceptive processing may contribute to behavioral avoidance of food and other salient stimuli in AN.

Contrary to our hypothesis and other studies suggesting that RAN have an elevated response during anticipation in the anterior insula^2,10^, we detected lower response during anticipation in RAN relative to CW that was localized to the ventral mid-insula. Anterior insula activation in healthy adults has typically been associated with anticipation of aversive stimuli, particularly when they are unpredictable^34,74,75^. This activation is further elevated in individuals either diagnosed or at risk for an anxiety disorder, suggesting heightened aversive anticipation reactivity may be associated with anxiety^76,77^. For our task, the anticipatory phase always predicted a pleasant touch rather than an aversive stimulus, and may have therefore failed to provoke prediction error by measuring neural anticipation under conditions of certainty. This reduced anticipatory signaling in RAN for predictable events likely confers an inability to effectively prepare for even certain upcoming experiences. Importantly, we found that RAN with lower BOLD responses during soft touch anticipation had higher harm avoidance scores, suggesting that anxiety and behavioral inhibition may play a role in disrupted interoception expectancy in AN. Similarly, others have found lower BOLD response in the left mid-insula to stomach interoceptive attention in both healthy controls and weight-restored AN is associated with greater levels of both clinical anxiety measures and harm avoidance^8^.

In comparison, both groups showed increased ventral mid-insula activation during soft touch receipt, but this response was greater in RAN relative to CW. These findings are consistent with the notion^34^ that the right anterior and mid-insula in particular may be more sensitive to stimuli that are generally arousing to the body. Affective touch stimuli is believed to reach the posterior insula via the spino-thalamo-cortical interoceptive pathway in humans and primates^11,78^, but in sub-primates and rodents may reach the forebrain via the spinoparabrachial pathway^79–82^. The posterior insula projects to the mid-insula, where inputs regarding affective stimuli are integrated with inputs from subcortical homeostatic control centers (i.e., hypothalamus and amygdala). This generates an integrated representation of both the internal and external environment, which is then projected to the anterior insula and integrated with input from cortical control regions (e.g., ventrolateral and dorsolateral prefrontal cortex) as well as regions involved with motivation and emotional salience (e.g., anterior cingulate cortex, orbitofrontal cortex, ventral striatum)^40,83^. The mid-insula thus responds not only to visceral input, but also to other domains, including exteroceptive and hedonic stimulation^34,42,58^, supporting its role in integrating salient features of the internal and external environment. Although ill AN have rated affective touch less pleasant than controls^84^, both groups rated pleasantness equally in our study. This is consistent with other studies where RAN rated the pleasantness, or unpleasantness, of stimuli similarly to CW^5,10,85,86^. A recent meta-analysis suggests both ill and remitted AN experience self-reported interoceptive deficits, which was more pronounced in the ill state and primarily reflected emotional awareness^87^. Notably, the RAN in our study reported soft touch as more intense, supporting a generalized finding of elevated intensity perception to somatosensory stimuli in both ill and weight-restored AN^27^. Notably, RAN who showed the most pronounced hyperactivation of the mid-insula also rated the experience as most intense, supporting altered modulation to sensory stimuli.

There were no significant differences in the insula related to touch location. Although the posterior insula has been associated with affective touch and may be more sensitive to C-fiber stimulation^88^, Aβ fibers can also produce a pleasant sensation in glabrous skin^89^ and have been shown to elicit BOLD responses in portions of the orbitofrontal cortex that are connected with the insula and are involved in emotional evaluation^90^. Moreover, a recent study in healthy volunteers suggests the insula processes both affective touch as well as discriminative touch^91^, supporting the lack of differentiation reported herein. Despite prior work showing that AN patients rate optimal velocity C-fiber stimulation as less pleasant than controls^84^, our study participants did not report differences in perceived pleasantness between soft touch to the palm and forearm, and both groups rated touch as equally pleasant. Studies in alcohol^47^ and substance^46,92^ users have also failed to show this palm-forearm distinction, suggesting the insula may play a role in both kinds of touch.

We also did not detect group-related activation differences within the striatum to either location or condition. Rather, we observed an overall greater BOLD response during soft touch receipt relative to anticipation in the dorsal striatum. This is consistent with the dorsal striatum’s role in reward processing, and likely reflects evaluation of soft touch receipt. The striatum is responsive to both the rewarding features of interoceptive stimuli that may serve as primary reinforcers, like taste, and to secondary rewards, such as money^93,94^ and social interactions^95^. A higher BOLD response during soft touch was also associated with greater pleasantness ratings in both groups, reflecting the striatum’s role in reward evaluation. The literature is mixed in terms of whether abnormalities of striatal function persist with remittance. Although some studies report abnormal striatal response to salient stimuli in remitted AN^5,9,96,97^, others suggest that the striatum’s response may be state dependent and largely normalizes with recovery^4,98,99^. Overall, these findings support the likelihood that the neural response to the anticipation and receipt of affective stimuli in the insula may be of particular importance in AN.

Our findings suggest that AN is characterized by a reduced responsivity to interoceptive anticipation in association with elevated levels of harm avoidance. Moreover, RAN individuals reporting touch as more intense also had lower BOLD responses during touch anticipation in both the insula and striatum, suggesting that an impaired ability to anticipate incoming stimuli may partly account for experiencing them more intensely. Poor prediction, coupled with elevated response to receipt, likely leads to the perception of a more intense experience. These findings are consistent with the notion that the difference between interoceptive accuracy and interoceptive confidence, or assurance in one’s interoceptive accuracy, predicts anxiety^100^ and may explain why AN individuals report low levels of trust in their interoceptive experience^101^. Our findings also suggest that the processing of arousal rather than valence of interoceptive experience may be aberrant in AN. This is further supported by neuroimaging findings of altered insula response during anticipation and receipt of both pleasant and aversive stimuli^2–5^, and evidence of a prolonged neural response to aversive breathing load^9^. RAN with greater body dissatisfaction also had a reduced response during touch anticipation, suggesting that impaired interoceptive processing may promote difficulty with self-assessment of one’s body. Others have reported inverse relationships between body satisfaction and impaired interoceptive awareness^102,103^. Together, these data provide further support that interoceptive prediction error may be of particular importance in the development and maintenance of AN^4,104–106^.

## Limitations

Although this represents the first neuroimaging study of affective touch in AN, our sample size was small, and future studies should include larger samples to explore moderators (anxiety, diagnostic subtype) on the processing of pleasant touch. It is not possible to determine whether findings are related to a trait of the disorder or are a consequence of malnutrition. RAN also did not endorse clinically significant levels of altered interoceptive awareness or body image disturbance. It is possible that those who achieve remittance may have had less disturbance than those who experience a more protracted, chronic course of illness. Additional studies are needed to determine whether the ill state is associated with greater disturbances of interoception in association with the BOLD response during soft touch. We did not assess for autistic traits; these have been associated with AN^107–109^, and future studies should examine these associations more closely. This study assessed anticipatory response to predictable and certain events. Given that AN experience increased prediction error response to unexpected events^4^ and an elevated intolerance of uncertainty^66^, the degree to which level of outcome predictability influences anticipatory response requires further investigation as this may account for differences across studies and have clinical implications. Inclusion of a continuous performance task and an anticipatory phase may have influenced our ability to detect meaningful differences related to C-fiber stimulation. Sensory information enters one’s subjective awareness based upon attention^110^ and other contextual information, such as visual stimuli or one’s internal motivational state^111–113^. Future studies should examine soft touch anticipation and receipt without a continuous performance task to detect potential differences in C-fiber stimulation. Finally, it is possible the presence of an individual applying the brush strokes may have given the interaction a social context, and future studies should ask participants about the experience of having someone administer the soft touch.

## Clinical implications

Results may have important implications for our understanding and effective treatment of AN. AN may suffer from impaired anticipation, coupled with subsequent altered processing of experienced physiological stimulation, which may in turn impact one’s sense of self. This may support body image disturbance and a desire to avoid affective and social stimuli, regardless of valence. Others have associated impaired interoception with increased anxiety;^24^ our data also support a relationship between elevated anxiety and a decreased anticipatory signal for pleasant stimuli. Given this mismatch between the anticipation and processing of internal and external stimuli, an intriguing question is whether these associations might be relearned^104,114^. Therapeutic approaches helping AN to better anticipate and interpret salient affective stimuli by increasing awareness of exteroceptive cues, and/or improved tolerance of expected and unexpected interoceptive experiences may be an important addition to current treatment approaches.

## Electronic supplementary material

Slow Stroke 26CWvs18RAN Supplemental 07 02 18
