# Supplementary material for: Neural hypersensitivity to pleasant touch in women remitted from anorexia nervosa
Source: Transl Psychiatry. 2018 Aug 16;8:161. doi: 10.1038/s41398-018-0218-3 (PMC6095886; doi:10.1038/s41398-018-0218-3)

Supplemental Table 1. Participant demographics and characteristics.

| **Characteristic** | **CW (N=26)** | **RAN (N=18)** | **Statistic** |
| --- | --- | --- | --- |
| Age (years) | 26.3 (7.4) | 26.3 (4.6) | t(1, 41.6)=0.0, p=1.0, d=0.0 |
| Current BMI | 21.7 (1.7) | 21.8 (2.2) | t(1, 30.7)=-0.0, p=1.0, d=-0.0 |
| Lowest BMI^a^ | 19.8 (1.4) | 15.0 (1.5) | t(1, 34.7)=10.8, p<0.001, d=3.3 |
| Education(Years)^b^ | 15.3 (1.4) | 15.8 (2.2) | t(1, 24.5)=-0.8, p=0.5, d=-0.2 |
| BDI | 0.8 (1.6) | 2.3 (2.5) | t(1, 26.5)=-2.2, p=0.03, d=-0.7 |
| EDI Drive for Thinness | 0.1 (0.4) | 1.1 (1.8) | t(1, 18.1)=-2.4, p=0.03, d=-0.7 |
| EDI Body Dissatisfaction | 0.6 (1.4) | 2.3 (2.9) | t(1, 22.6)=-2.3, p=0.03, d=-0.7 |
| EDI Ineffectiveness | 0.5 (1.0) | 0.9 (1.2) | t(1, 33.3)=-1.2, p=0.2, d=-0.4 |
| EDI Perfectionism | 4.9 (3.5) | 8.8 (4.0) | t(1, 33.5)=-3.4, p<0.001, d=-1.0 |
| EDI Interpersonal Distrust | 0.7 (1.5) | 1.4 (1.9) | t(1, 31.1)=-1.4, p=0.2, d=-0.4 |
| EDI Interoceptive Awareness | 0.2 (0.7) | 0.7 (2.6) | t(1, 18.7)=-0.8, p=0.5, d=-0.2 |
| State Anxiety | 22.9 (2.3) | 28.4 (5.1) | t(1, 21.7)=-4.3, p<0.001, d=-1.3 |
| Trait Anxiety | 23.6 (2.9) | 30.6 (6.9) | t(1, 21.2)=-4.0, p<0.001, d=-1.2 |
| TCI Harm Avoidance^a^ | 7.3 (4.4) | 13.2 (7.1) | t(1, 26.1)=-3.1, p<0.001, d=-0.9 |
| Y-BOCS Current Total^a^ | 0.0 (0.0) | 0.0 (0.0) | N/A |
| Y-BOCS Whole Lifetime Total^a^ | 0.0 (0.0) | 1.9 (5.1) | t(1, 17.0)=-1.6, p=0.1, d=-0.5 |
| Illness Duration (months) | N/A | 10.3 (8.9) |  |
| Months Since Last Symptoms^b^ | N/A | 53.6 (48.6) |  |
| Lowest BMI | N/A | 15.0 (1.5) |  |
| Note: Entries are of the form mean (standard deviation). Statistical comparisons were by means of Welch’s t-tests, and Cohen’s *d* effect sizes are reported. BDI-II: Beck Depression Inventory II; BMI: body mass index; CW: healthy comparison women; N/A: not applicable; EDI: Eating Disorders Inventory; RAN: women remitted from anorexia nervosa; STAI: Spielberger State-Trait Anxiety Inventory; TCI: Temperament and Character Inventory; Y-BOCS: Yale-Brown Obsessive Compulsive Scale. ^a^One CW did not complete this assessment. ^b^One RAN did not complete this assessment. | | | |

Supplemental Table 2. VAS ratings on the experience of soft touch of the forearm or palm.

|  | **CW^a^** | **RAN** | **Statistic** |
| --- | --- | --- | --- |
| Pre-Scan Forearm |  |  |  |
| Pleasant | 48.9 (26.6) | 47.8 (19.3) | t(1, 41.9)=0.16, p=0.87, d=0.05 |
| Unpleasant | 7.8 (15.6) | 5.8 (10.6) | t(1, 42.0)=0.49, p=0.63, d=0.15 |
| Intense | 6.9 (14.6) | 5.7 (7.7) | t(1, 39.8)=0.34, p=0.73, d=0.11 |
| Post-Scan Forearm |  |  |  |
| Pleasant | 47.1 (26.6) | 46.2 (24.2) | t(1, 38.7)=0.12, p=0.90, d=0.04 |
| Unpleasant | 4.7 (10.6) | 10.9 (20.0) | t(1, 23.8)=-1.19, p=0.24, d=-0.37 |
| Intense | 5.4 (12.7) | 13.9 (22.5) | t(1, 24.8)=-1.44, p=0.16, d=-0.44 |
| Pre-Post Scan Forearm |  |  |  |
| Pleasant | 3.6 (19.5) | 1.6 (17.4) | t(1, 39.0)=0.34, p=0.73, d=0.11 |
| Unpleasant | 2.4 (16.5) | -5.1 (14.7) | t(1, 39.0)=1.56, p=0.13, d=0.48 |
| Intense | 1.7 (13.8) | -8.2 (20.2) | t(1, 28.2)=1.79, p=0.08, d=0.55 |
| Pre-Scan Palm |  |  |  |
| Pleasant | 50.3 (21.6) | 45.1 (18.5) | t(1, 40.0)=0.85, p=0.40, d=0.26 |
| Unpleasant | 6.4 (14.4) | 4.9 (8.5) | t(1, 41.1)=0.44, p=0.66, d=0.14 |
| Intense | 7.5 (15.4) | 13.7 (25.5) | t(1, 25.6)=-0.93, p=0.36, d=-0.29 |
| Post-Scan Palm |  |  |  |
| Pleasant | 51.2 (25.0) | 44.5 (22.9) | t(1, 38.6)=0.90, p=0.37, d=0.28 |
| Unpleasant | 2.9 (5.9) | 10.6 (20.4) | t(1, 19.1)=-1.55, p=0.14, d=-0.48 |
| Intense | 3.1 (7.8) | 17.7 (23.8) | t(1, 19.6)=-2.51, p=0.02, d=-0.78 |
| Pre-Post Scan Palm |  |  |  |
| Pleasant | -0.9 (20.2) | 0.6 (18.7) | t(1, 38.3)=-0.25, p=0.80, d=-0.08 |
| Unpleasant | 3.4 (16.0) | -5.7 (18.3) | t(1, 33.7)=1.68, p=0.10, d=0.52 |
| Intense | 4.7 (15.6) | -3.9 (30.6) | t(1, 23.4)=1.10, p=0.28, d=0.34 |

Note: Entries are of the form mean (standard deviation). Statistical comparisons were by means of Welch’s t-tests, and Cohen’s *d* effect sizes are reported. CW: healthy comparison women RAN: women remitted from anorexia nervosa; ^a^One CW failed to complete the post-scan VAS ratings.

| Supplemental Table 3. Voxelwise linear mixed effects analysis results for the soft touch paradigm showing main effects of Condition (Anticipation, Soft Touch) and Location (Palm, Forearm), and interactions of Group (CW, RAN) x Condition. | | | | | | | | | | |  |
| --- | --- | --- | --- | --- | --- | --- | --- | --- | --- | --- | --- |
|  | | | | | | | **Post Hoc Comparisons** | | | |  |
| **Region** | **L/R** | **Volume (voxels)** | **x** | **y** | **z** | **F value** | | **Comparison** | **z** | **p(FDR)** | |
| **CONDITION*** | | | | | | | | | | |  |
| Cingulate Gyrus | L | 1960 | -21 | -22 | 31 | 51.59 | | Soft Touch > Anticipation | 6.363 | <0.001 | |
| Lentiform Nucleus | R | 893 | 24 | -11 | 10 | 39.73 | | Soft Touch > Anticipation | 5.676 | <0.001 | |
| Superior Temporal Gyrus | R | 615 | 52 | -34 | 9 | 36.86 | | Soft Touch > Anticipation | 6.465 | <0.001 | |
| Inferior Frontal Gyrus | L | 377 | -44 | 17 | 1 | 29.88 | | Soft Touch > Anticipation | 5.989 | <0.001 | |
| Cuneus | R | 318 | 12 | -74 | 12 | 31.37 | | Soft Touch > Anticipation | 5.154 | <0.001 | |
| Middle Frontal Gyrus | L | 242 | -20 | 29 | 46 | 25.5 | | Soft Touch > Anticipation | 5.513 | <0.001 | |
| Medial Frontal Gyrus | R | 180 | 17 | 33 | 47 | 25.19 | | Soft Touch > Anticipation | 5.882 | <0.001 | |
| Precentral Gyrus | R | 138 | 43 | -12 | 36 | 26.65 | | Soft Touch > Anticipation | 5.599 | <0.001 | |
| Precuneus | R | 124 | 1 | -54 | 31 | 18.55 | | Soft Touch > Anticipation | 4.313 | <0.001 | |
| Inferior Frontal Gyrus | L | 71 | -51 | 17 | 17 | 33.6 | | Soft Touch > Anticipation | 6.054 | <0.001 | |
| Middle Temporal Gyrus | L | 70 | -56 | -47 | 2 | 20.76 | | Soft Touch > Anticipation | 5.029 | <0.001 | |
| Supramarginal Gyrus | R | 58 | 47 | -58 | 33 | 21.26 | | Soft Touch > Anticipation | 5.022 | <0.001 | |
| Inferior Frontal Gyrus | R | 53 | 38 | 38 | 10 | 23.15 | | Soft Touch > Anticipation | 4.721 | <0.001 | |
| Inferior Parietal Lobule | R | 48 | 44 | -38 | 39 | 23.91 | | Soft Touch > Anticipation | 4.547 | <0.001 | |
| Inferior Frontal Gyrus | R | 45 | 36 | 34 | -7 | 26.95 | | Soft Touch > Anticipation | 5.312 | <0.001 | |
| Inferior Parietal Lobule | L | 27 | -55 | -36 | 39 | 16.91 | | Soft Touch > Anticipation | 4.401 | <0.001 | |
| Superior Frontal Gyrus | L | 26 | -9 | 59 | 26 | 19.35 | | Soft Touch > Anticipation | 4.686 | <0.001 | |
| Inferior Parietal Lobule | R | 25 | 44 | -50 | 59 | 24.23 | | Soft Touch > Anticipation | 5.365 | <0.001 | |
| Middle Frontal Gyrus | R | 18 | 24 | -14 | 64 | 24.12 | | Anticipation > Soft Touch | 4.489 | <0.001 | |
| Inferior Parietal Lobule | L | 17 | -50 | -48 | 40 | 18.57 | | Soft Touch > Anticipation | 4.173 | <0.001 | |
| Parahippocampal Gyrus | L | 12 | -25 | -37 | 0 | 20.41 | | Soft Touch > Anticipation | 4.175 | <0.001 | |
| **LOCATION** | | | | | | | | | | |  |
| Postcentral Gy | R | 218 | 41 | -22 | 52 | 37.27 | | Palm > Forearm | 5.256 | <0.001 | |
| **GROUP x CONDITION** | | | | | | | | | | |  |
| Ventral Insula/Superior Temporal Gyrus | R | 114 | 44 | -13 | -9 | 15.92 | | CW: Soft Touch > Anticipation | 2.97 | 0.018 | |
|  |  |  |  |  |  |  | | Soft Touch: RAN > CW | 2.67 | 0.046 | |
|  |  |  |  |  |  |  | | RAN: Soft Touch > Anticipation | 6.86 | <0.001 | |
| Note: Center of mass coordinates reported in MNI space. Correction for multiple comparisons was determined with Monte-Carlo simulations (via AFNI’s 3dClustSim) to guard against false positives. Post hoc analyses were conducted using glht from the multcomp package in R to calculate general linear hypotheses using Tukey’s all-pair comparisons, and p-values were Bonferroni adjusted. CW: healthy comparison women; L: left; R: right; RAN: women remitted from anorexia nervosa. *Because a main effect of condition encompassed nearly the whole brain, a more stringent threshold of p<0.0005 (minimum cluster size 7 voxels) was used to better describe effects therein. | | | | | | | | | | |  |

| Supplemental Table 4. Significant clusters identified by exploratory robust regression analyses associating brain activity with VAS ratings and clinical measures within the insula and striatum. | | | | | | | | | |
| --- | --- | --- | --- | --- | --- | --- | --- | --- | --- |
| **Event** | **Region** | **L/R** | **Volume (voxels)** | **X** | **Y** | **Z** | **t** | **r** | **p** |
| **CW: Pre-Scan Pleasant** | | | | | | | | | |
| Anticipation Forearm | Caudate | L | 11 | -9 | 13 | 13 | 3.52 | 0.54 | 0.004 |
| Anticipation Palm | Caudate | R | 61 | 12 | 15 | -2 | 3.52 | 0.62 | 0.001 |
|  | Putamen | L | 57 | -14 | 12 | -6 | 4.04 | 0.65 | <0.001 |
| **RAN: Pre-Scan Pleasant** | | | | | | | | | |
| Soft Touch Forearm | Putamen | R | 20 | 25 | 5 | -3 | 5.91 | 0.81 | <0.001 |
|  | Caudate | L | 10 | -11 | 22 | 6 | 4.06 | 0.6 | 0.009 |
|  |  | R | 12 | 12 | 0 | 18 | 3.55 | 0.7 | 0.001 |
| Soft Touch Palm | Caudate | L | 10 | -17 | -17 | 24 | 3.24 | 0.66 | 0.003 |
|  |  | R | 37 | 16 | -1 | 23 | 4.85 | 0.69 | 0.002 |
| **CW: Pre-Scan Intense** | | | | | | | | | |
| None |  |  |  |  |  |  |  |  |  |
| **RAN: Pre-Scan Intense** | | | | | | | | | |
| Palm | Putamen | R | 14 | 23 | 0 | 6 | 3.43 | 0.67 | 0.002 |
| **CW: Post-Scan Pleasant** | | | | | | | | | |
| Soft Touch Forearm | Caudate | L | 10 | -14 | 22 | -1 | 4.45 | 0.41 | 0.04 |
| **RAN: Post-Scan Pleasant** | | | | | | | | | |
| Soft Touch Palm | Caudate | L | 15 | -17 | -15 | 24 | 3.3 | 0.71 | 0.001 |
| **CW: Post-Scan Intense** | | | | | | | | | |
| Anticipation Palm | Dorsal Anterior Insula | L | 28 | -31 | 17 | 4 | 3.27 | 0.59 | 0.002 |
|  |  | R | 29 | 35 | 19 | 1 | 5 | 0.55 | 0.005 |
| Soft Touch Palm | Ventral Mid-Insula | L | 38 | -36 | 12 | -10 | 5.02 | 0.69 | <0.001 |
|  | Dorsal Mid-Insula |  | 21 | -39 | -6 | 6 | 3.7 | 0.62 | 0.001 |
|  | Dorsal Mid-Insula |  | 11 | -28 | 18 | 9 | 3.37 | 0.58 | 0.003 |
|  | Posterior Insula | R | 14 | 38 | -9 | 7 | 3.6 | 0.59 | 0.002 |
|  | Caudate | R | 12 | 12 | 13 | 15 | 3.31 | 0.53 | 0.007 |
| **RAN: Post-Scan Intense** | | | | | | | | | |
| Anticipation Forearm | Posterior Insula | L | 30 | -34 | -18 | 6 | -4.73 | -0.74 | <0.001 |
|  | Anterior Ventral Insula | R | 10 | 33 | 12 | -15 | -5.6 | -0.57 | 0.013 |
|  | Caudate | L | 30 | -11 | 18 | 5 | -3.44 | -0.67 | 0.002 |
|  |  |  | 9 | -9 | 2 | 10 | -3.63 | -0.6 | 0.008 |
|  |  |  | 9 | -13 | 1 | 24 | -3.71 | -0.72 | 0.001 |
|  |  | R | 86 | 12 | 9 | 13 | -5.29 | -0.69 | 0.001 |
|  | Putamen | L | 28 | -28 | -9 | 5 | -4.42 | -0.55 | 0.019 |
| Soft Touch Palm | Ventral Mid-Insula | L | 21 | -38 | -8 | 5 | 3.32 | 0.6 | 0.008 |
|  | Dorsal Mid-Insula | L | 19 | -32 | 13 | -8 | 4.32 | 0.76 | <0.001 |
|  |  |  | 17 | -38 | -12 | -8 | 3.8 | 0.71 | 0.001 |
|  | Putamen | L | 14 | -26 | 2 | -4 | 2.99 | 0.64 | 0.004 |
| **RAN: Lowest BMI** | | | | | | | | | |
| Soft Touch Forearm | Ventral Anterior Insula | R | 15 | 36 | 11 | -14 | 4.88 | 0.72 | 0.001 |
|  | Dorsal Mid-Insula | R | 10 | 43 | -3 | 4 | -4.82 | -0.68 | 0.002 |
| **RAN: Illness Duration** | | | | | | | | | |
| Soft Touch Forearm | Ventral Anterior Insula | L | 8 | -37 | 13 | -11 | 3.95 | 0.7 | 0.001 |
| **RAN: Body Dissatisfaction** | | | | | | | | | |
| Soft Touch Forearm | Ventral Mid-Insula | R | 17 | 43 | 2 | -2 | -5.02 | -0.63 | 0.006 |
| Note: Coordinates are reported as the center of mass. CW: healthy comparison women; L: left; R: right; RAN women remitted from anorexia nervosa. | | | | | | | | | |

Supplemental Figure 1. RAN [t=-5.02, p=0.006] with higher body dissatisfaction had lower BOLD response during anticipation of touch of the forearm in the right ventral mid-insula, as identified by Huber robust regression. RAN: women remitted from anorexia nervosa; L: left; R: right; EDI: Eating Disorders Inventory-2. *p<0.05; ***p<0.005


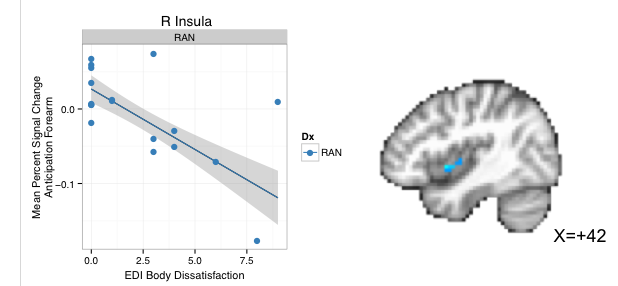

Supplement: Supplementary file 1 — Slow Stroke 26CWvs18RAN Supplemental 07 02 18 [file 41398_2018_218_MOESM1_ESM.docx]
